# Koch and His Discovery

**Published:** 1891-05

**Authors:** C. B. Gilbert

**Affiliations:** Washington, D. C.


### KOCH AND HIS DISCOVERY.

C. B. Gilbert, M. D., Washington, D. C.

There is so much interest in "Koch's discovery," and there has recently been so much excitement over Pasteur's treatment of hydrophobia that it seems fitting that honor should be given to whom honor is due: First, Jenner, the discoverer of vaccination, from which discovery all these later ones have sprung; next, to the late Dr. Constantine Hering, of Philadelphia, who in 1830 directed, in *Stapf's Archives*, how to prepare virus from anthrax; it was done by Dr. G. A. Weber, who cured with it every case in cattle and the herders who had contracted the disease. His report was published in Leipzig in 1836, but no notice was taken of it except by Dr. P. Dufresne, of Geneva, who used the prepared virus and prevented the further spread of the murderous disease among the sheep and shepherds. His report was published in Geneva in January and February, 1837. Dr. Hering says that "the discovery of the bacteria and their incredibly rapid propagation seemed to be of much more importance than the cure of cattle and the loss of millions of dollars by this disease. Radiant heat, proposed scores of years ago [this was written prior to 1879. C. B. G.] for other zymotic diseases by C. Hering, was discovered in a very ingenious way by Pasteur, to prevent the increase of bacteria. Now, the heat (as it has done in hydrophobia) and the nosode [disease products. C. B. G.] may suffice to cure every case. Dr. Kasemann had moral courage enough to introduce anthracine in gangrene in 1853, and Dr. Raue has given it in carbuncles since 1858." The last use can be corroborated in every part of the United States.

In 1830 Dr. Hering, after having, in South America, experimented upon himself with snake poison, wrote as follows: "The proving of snake poison may pave the way to the prevention of hydrophobia and of variola by the proving of the respective morbific poisons." This was repeated in 1833. In August, 1833, he procured some saliva from a rabid dog, prepared it by triturating with milk sugar, and then preserved it in alcohol. With this preparation in different strengths experiments were made on the healthy (provings), and later many cures were made of conditions similar to those produced upon the provers. Dr. Hering says that of many persons who, having been bitten, took Lyssin, as the prepared virus is called, none ever developed hydrophobia. This is negative testimony, of course, but just as good as Pasteur's after his treatment.

Dr. Hering did not find it necessary to dilute his virus through the spinal marrow of rabbits, or in any other elaborate or painful method, but in a simple way with non-medicinal vehicles.

Dr. Koch has yet to demonstrate that tubercle bacillus causes tuberculosis, and that he can cure with his preparation incipient tuberculosis or even lupus ; if he can, all honor to him, but let us honor the pioneers even though they be not members of academies of science.—*Washington Evening Star*.

